# The correlation of hemoglobin and 28-day mortality in septic patients: secondary data mining using the MIMIC-IV database

**DOI:** 10.1186/s12879-023-08384-9

**Published:** 2023-06-20

**Authors:** Yu Chen, Lu Chen, Zengping Meng, Yi Li, Juan Tang, Shaowen Liu, Li Li, Peisheng Zhang, Qian Chen, Yongmei Liu

**Affiliations:** 1 Clinical Laboratory Center, The Affiliated Hospital of Guizhou Medical University, 28, Guiyi Street, Guiyang, Guizhou China; 2 Department of Clinical Trials Centre, The Affiliated Hospital of Guizhou Medical University, 28, Guiyi Street, Guiyang, Guizhou China; 3 College of Medical Laboratory, Guizhou Medical University, 9 Beijing Road, Guiyang, Guizhou China

**Keywords:** Hemoglobin, sepsis, Retrospective study, MIMIC-IV

## Abstract

**Background:**

Previous studies found minimal evidence and raised controversy about the link between hemoglobin and 28-day mortality in sepsis patients. As a result, the purpose of this study was to examine the association between hemoglobin and 28-day death in sepsis patients by analyzing the Medical Intensive Care IV (MIMIC-IV) database from 2008 to 2019 at an advanced medical center in Boston, Massachusetts.

**Methods:**

We extracted 34,916 sepsis patients from the MIMIC-IV retrospective cohort database, using hemoglobin as the exposure variable and 28-day death as the outcome variable, and after adjusting for confounders (demographic indicators, Charlson co-morbidity index, SOFA score, vital signs, medication use status (glucocorticoids, vasoactive drugs, antibiotics, and immunoglobulins, etc.)), we investigated the independent effects of hemoglobin and 28-day risk of death by binary logistic regression as well as two-piecewise linear model, respectively.

**Results:**

Hemoglobin levels and 28-day mortality were shown to be non-linearly related.The inflection points were 104 g/L and 128 g/L, respectively. When HGB levels were between 41 and 104 g/L, there was a 10% decrease in the risk of 28-day mortality (OR: 0.90; 95% CI: 0.87 to 0.94, p-value = 0.0001). However, in the range of 104–128 g/L, we did not observe a significant association between hemoglobin and 28-day mortality (OR: 1.17; 95% CI: 1.00 to 1.35, P value = 0.0586). When HGB was in the range of 128–207 g/L, there was a 7% increase in the risk of 28-day mortality for every 1 unit increase in HGB (OR: 1.07; 95% CI: 1.01 to 1.15, P value = 0.0424).

**Conclusion:**

In patients with sepsis, baseline hemoglobin was related to a U-shaped risk of 28-day death. When HGB was in the range of 12.8–20.7 g/dL, there was a 7% increase in the risk of 28-day mortality for every 1 unit increase in HGB.

## Background

Sepsis is now defined as a syndrome of poor host response to an infectious illness, followed by a succession of life-threatening organ dysfunctions. Sepsis is one of the leading causes of inpatient death globally, and its prevalence has gradually grown in recent years [1, 2]. According to epidemiological data, one million individuals with sepsis are hospitalized in the United States each year, with a death rate of up to 20%. A recent study based on adult hospitalization data from seven high-income nations found a 19.4 million yearly incidence of sepsis and nearly 5.3 million patient fatalities related to sepsis.

Unfortunately, despite significant efforts to discover novel medications and study the etiology of sepsis, mortality from sepsis has not improved in the 14 years from 2002 to 2016. The bottleneck is that there are no sufficiently reliable models that reliably forecast sepsis prognosis and so give a foundation for prognostic enrichment, leaving clinical trials of novel medications with insufficient power.However, clarifying the real link between the variables in the model and the outcome is a precondition for excellent performance prediction models.Hemoglobin is the exposure variable of interest in this article.

Hemoglobin is an intravascular macromolecular material that is generally maintained in human blood circulation. Hemoglobin is also important in the defense against pathogens and in the function of white blood cells. It promotes non-specific immunity by chelating iron [3] and thereby denying bacteria of nutrition, processing carbon dioxide, maintaining hemodynamic stability, and absorbing and transporting antibiotics [4].

Different levels of hemoglobin concentrations in sepsis patients may be due to presenting conditions [4], nutritional deficiencies, inflammatory anemia [5–7], hemolysis [8, 9], loss of bloodletting, loss of gastrointestinal tract, transfusion expansion, fluid load resulting in hemodilution [10], decreased bone marrow response to erythropoietin [11], inhibition of erythropoietin production by pro-inflammatory cytokines [7, 12], response to drugs [13], inappropriate blood transfusion [14] and so on.

Little is known regarding the predictive usefulness of hemoglobin in ICU(surgical intensive care unit) patients with sepsis. Clinical research is now focusing on the link between anemia markers such as erythrocyte distribution width [15] and platelets [5, 13, 16, 17] and sepsis prognosis. Sepsis patients frequently have anemia, and a few studies have found that hemoglobin levels equal to or below 8 g/dL in the first 48 h after ICU admission are associated with a higher risk of death from sepsis [18, 19]. However, there is still debate about the importance of higher hemoglobin levels in contributing to a better prognosis in these patients. It is unknown which hemoglobin levels give the best prognosis for sepsis patients [9, 20, 21].

As a result, this study aims to investigate the relationship between hemoglobin and the 28-day risk of mortality from sepsis using the MIMIC-IV database for sepsis in a large US sample. The enormous sample size and sensitivity analysis of numerous databases in diverse populations will allow us to deliver more consistent and accurate results, allowing us to get a better understanding of the relationship between hemoglobin and the 28-day risk of mortality in sepsis.

## Methods

### Description of data sources

The Medical Information Mart for Intensive Care IV (MIMIC-IV) database was utilized to gather the data for this investigation. The MIMIC-IV database was built on the successful implementation of MIMIC-III [15]. It addresses the shortcomings of the MIMIC-III database, which was intended as a retrospective cohort study. It gathered clinical data on patients who visited Beth Israel Deaconess Medical Center(BIDMC) between 2008 and 2019. The database is free to download after completing an authorized course on their official website. The author, Lu Chen, has completed the accredited course, has access to the database, and is in charge of data extraction(Record ID: 28,572,693). Our work adheres to the RECORD (Research Reports Using Routine Collection of Observational Health Data) declaration.

### Cohort information

The MIMIC-IV database, containing a total of 377,207 adult patient records, was used in this study. From this population, we extracted 35,010 individuals diagnosed with sepsis using the ICD code information in the database. Specifically, patients were screened based on the following sepsis-related ICD codes: ICD-9 codes (99,591–99,592) and ICD-10 codes (R652, R6520 and R6521).The extraction phase lasted from 2008 to 2019 saw these patients in BIDMC. We looked at the relationship between 28-day death as the outcome variable (binary variable, Y = 1, death; Y = 0, survival) and basal hemoglobin as the exposure variable (recorded as a continuous variable). The hemoglobin measurement taken on the first day of admission to the ICU is defined as basal hemoglobin.Patients with missing exposure variable information were not included in this research.

The following covariates were chosen: demographic factors: gender (male or female), age (years), ethnicity, Charlson comorbidity index, SOFA (Sequential Organ Failure Assessment) score, APACHE(Acute Physiology And Chronic Health Evaluation) score, use of mechanical ventilation, use of glucocorticoids (dexamethasone, methylprednisolone, hydrocortisone), use of vasoactive drugs (dopamine,dobutamine), use of immunoglobulins, antibiotics (capreonan, cephalosporins, penicillin antibiotics, and vancomycin), heart rate and respiratory rate within the first hour of admission, lactate, heart failure, blood transfusions, gastrointestinal bleeding (GI), chronic ostructive pulmonary disease (COPD), ischemic heart disease, obstructive sleep apnea (OSA) and body temperature within 24 h of admission. The rationale for selecting these factors was mostly based on our clinical experience and the literature [15, 22].

### Statement of ethics and informed consent

For this study, the MIMIC-IV database was downloaded from the Internet. The Institutional Review Boards of Beth Israel Deaconess Medical Center in Boston, Massachusetts (2001-P-001699/14) and the Massachusetts Institute of Technology approved this study (0403000206).This study was approved by the Institutional Review Board (IRB) of the Affiliated Hospital of Guizhou Medical University and deemed exempt from the requirement for ethical approval.Through the Protection of Human Research Participants Examination, the authors have the right to use and download the database. Given that the data is public and patient identifiable information is unknown, informed patient permission was revoked.

### Missing data description

The variables used in this study were missing at a rate of less than 5% (0-4.1%), therefore, multiple interpolation was not used for missing data.

### Statistical analysis

Continuous variables are represented by the mean, standard deviation (normal distribution), or median (minimum, maximum) (skewed distribution). Categorical variables are expressed as percentages or frequencies. Given that this was a cohort study, we divided the exposure variables into 4 groups and examined the distribution of patient baseline information across the various subgroups. To evaluate any statistical differences between the means and proportions of the groups, one-way ANOVA (normal distribution), Kruskal-Wallis H (skewed distribution) tests, and chi-square tests (categorical variables) were performed. We also looked at the relationship between hemoglobin and 28-day mortality in septic patients using univariate and multivariate binary logistic regression models.We present unadjusted models (no covariates adjusted, Model 1), minimally-adjusted models (adjusted for demographic factors only, Model 2), and fully-adjusted models (adjusted for all covariates presented in Table 1, Model 3) with 95% confidence intervals, as well as the ratio (OR) with 95% confidence intervals.To improve the robustness of our results, we initially categorized hemoglobin levels and performed a trend test to determine its consistency as a continuous or categorical variable. Next, we performed a stratified analysis using variables related to hemoglobin levels as stratification variables to evaluate the consistency of hemoglobin with respect to risk of death within 28 days across different populations, and to test for interaction using a log-likelihood ratio test. Because hemoglobin is a continuous variable, a non-linear relationship cannot be ruled out. Given the limitations of binary logistic regression models to handle non-linear correlations, we investigated the relationship between hemoglobin and 28-day mortality in sepsis patients using a generalized additive model (GAM) and a smoothed curve fit. If there was a non-linear relationship, we used a recursive algorithm to calculate the inflection point value, and then a two-piecewise linear model to calculate OR values and 95% confidence intervals on either side of the inflection point.

All analyses were carried out using the statistical software packages R (http://www.R-project.org, The R Foundation)and EmpowerStats (http://www.empowerstats.com, X&Y Solutions, Inc, Boston, MA), with P values less than 0.05 (two-sided) deemed statistically significant.

## Results

### Description of the patient screening process

**Fig. 1:**
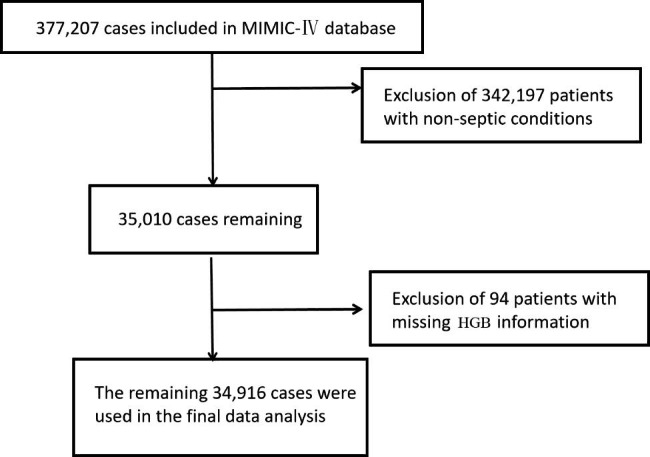
Patient selection process

This research initially enrolled 377,207 patients. 342,197 individuals were eliminated because they did not match the diagnostic criteria for sepsis, and 94 patients had missing HGB information, leaving a final balance of 34,916 patients for the final data analysis. The flow chart provides further information (Fig. 1).

### Patient baseline characteristics

Table 1 presents baseline characteristics of the patients, stratified into four groups (Q1-Q4) based on quadratic grouping of hemoglobin levels. We observed trends in variable distribution among the subgroups after grouping. The mean age of the patients was (66.67 ± 16.01) years, and 28-day mortality (Y = 1 for death) was 16.23% (5,668/34,926). In comparison to the Q4 group, the Q1, Q2, and Q3 groups had patients who were older, had a higher proportion of Caucasians, had higher Charlson co-morbidity index, SOFA score, and APACHE III score, and were more likely to have received dopamine, immunoglobulin, methylprednisolone, hydrocortisone, carbapenems, cephalosporins, cilins, blood transfusions, and vancomycin. These groups also had a higher rate of OSA, heart failure, GI bleeding, COPD, and ischemic heart disease. Additionally, we noticed that the Q1, Q2, and Q3 groups had lower body temperatures compared to the Q4 group. Similarly, we observed a lower proportion of patients in the Q2 and Q3 groups in terms of heart rate, respiratory rate, use of dexamethasone and dobutamine, and mechanical ventilation, as compared to the Q4 group.

**Table 1:** Description of baseline characteristics of patients with sepsis in the MIMIC-IV database

Variables	Q1(4.10–8.90)	Q2(8.90-10.15)	Q3(10.20–11.70)	Q4(11.75–20.70)	P-value
	N = 8729	N = 8553	N = 8905	N = 8729	
Socio-demographic variables					
Age at admission, mean ± sd, year	66.82 ± 15.54	68.38 ± 14.78	67.56 ± 15.84	63.96 ± 17.37	< 0.001
Ethnicity, n (%)					< 0.001
White	5813 (66.59%)	5967 (69.76%)	6055 (68.00%)	5770 (66.10%)	
Other	2916 (33.41%)	2586 (30.24%)	2850 (32.00%)	2959 (33.90%)	
Gender					< 0.001
Male	4634 (53.09%)	4500 (52.61%)	5081 (57.06%)	5925 (67.88%)	
Female	4095 (46.91%)	4053 (47.39%)	3824 (42.94%)	2804 (32.12%)	
Vital sign variables					
Heart rate, mean ± sd, times/minute	105.09 ± 24.59	102.99 ± 24.01	103.15 ± 24.31	104.59 ± 25.06	< 0.001
Number of breaths, mean ± sd, times/minute	27.49 ± 9.74	26.19 ± 9.74	26.36 ± 9.63	26.75 ± 9.44	< 0.001
Body temperature, mean ± sd, ℃	36.77 ± 1.25	36.63 ± 1.28	36.75 ± 1.26	36.94 ± 1.27	< 0.001
Indicators related to disease severity					
SOFA score, mean ± sd, score	7.34 ± 3.98	6.70 ± 3.66	6.24 ± 3.56	5.99 ± 3.56	< 0.001
APACHE III score, mean ± sd, score	78.44 ± 27.56	72.47 ± 26.94	68.59 ± 26.93	65.57 ± 27.10	< 0.001
Lactate, median (min, max) mmol/L	2.10 (0.20–29.60)	2.30 (0.00-28.70)	2.20 (0.40–23.80)	2.20 (0.30–89.00)	< 0.001
Co-morbidity related variables					
Charlson comorbidity index, mean ± sd, score	6.92 ± 2.95	6.46 ± 2.82	5.90 ± 2.82	5.20 ± 2.88	< 0.001
Heart failure, n (%)					< 0.001
No	4694 (53.77%)	4771 (55.78%)	5433 (61.01%)	5858 (67.11%)	
Yes	4035 (46.23%)	3782 (44.22%)	3472 (38.99%)	2871 (32.89%)	
Gastrointestinal bleeding, n (%)					< 0.001
No	8065 (92.39%)	8152 (95.31%)	8637 (96.99%)	8598 (98.50%)	
Yes	664 (7.61%)	401 (4.69%)	268 (3.01%)	131 (1.50%)	
Chronic Obstructive Pulmonary Disease, n (%)					< 0.001
No	7592 (86.97%)	7723 (90.30%)	8069 (90.61%)	7927 (90.81%)	
Yes	1137 (13.03%)	830 (9.70%)	836 (9.39%)	802 (9.19%)	
Ischemic heart disease, n (%)					< 0.001
No	6510 (74.58%)	6492 (75.90%)	7009 (78.71%)	7138 (81.77%)	
Yes	2219 (25.42%)	2061 (24.10%)	1896 (21.29%)	1591 (18.23%)	
Obstructive sleep apnea, n (%)					0.151
No	7406 (84.84%)	7337 (85.78%)	7540 (84.67%)	7402 (84.80%)	
Yes	1323 (15.16%)	1216 (14.22%)	1365 (15.33%)	1327 (15.20%)	
Treatment-related variables					
Glucocorticoid use					
Dexamethasone use, n(%)					< 0.001
No	7589 (86.94%)	7719 (90.25%)	8062 (90.53%)	7736 (88.62%)	
Yes	1140 (13.06%)	834 (9.75%)	843 (9.47%)	993 (11.38%)	
Methylprednisolone use, n (%)					< 0.001
No	6836 (78.31%)	7015 (82.02%)	7465 (83.83%)	7467 (85.54%)	
Yes	1893 (21.69%)	1538 (17.98%)	1440 (16.17%)	1262 (14.46%)	
Hydrocortisone use, n (%)					< 0.001
No	8503 (97.41%)	8351 (97.64%)	8749 (98.25%)	8589 (98.40%)	
Yes	226 (2.59%)	202 (2.36%)	156 (1.75%)	140 (1.60%)	
Vasoactive agents					
Dopamine use, n(%)					0.012
No	8070 (92.45%)	7921 (92.61%)	8302 (93.23%)	8167 (93.56%)	
Yes	659 (7.55%)	632 (7.39%)	603 (6.77%)	562 (6.44%)	
Dobutamine use, n(%)					0.006
No	8300 (95.09%)	8195 (95.81%)	8561 (96.14%)	8362 (95.80%)	
Yes	429 (4.91%)	358 (4.19%)	344 (3.86%)	367 (4.20%)	
Antibiotic use					
Use of carbapenems, n (%)					< 0.001
No	5978 (68.48%)	6602 (77.19%)	7329 (82.30%)	7571 (86.73%)	
Yes	2751 (31.52%)	1951 (22.81%)	1576 (17.70%)	1158 (13.27%)	
Cephalosporin administration, n (%)					< 0.001
No	7880 (90.27%)	7767 (90.81%)	8179 (91.85%)	8068 (92.43%)	
Yes	849 (9.73%)	786 (9.19%)	726 (8.15%)	661 (7.57%)	
Penicillin antibiotics use, n (%)					< 0.001
No	3581 (41.02%)	4124 (48.22%)	4581 (51.44%)	4575 (52.41%)	
Yes	5148 (58.98%)	4429 (51.78%)	4324 (48.56%)	4154 (47.59%)	
Vancomycin administration, n (%)					< 0.001
No	968 (11.09%)	1525 (17.83%)	1957 (21.98%)	2086 (23.90%)	
Yes	7761 (88.91%)	7028 (82.17%)	6948 (78.02%)	6643 (76.10%)	
Supportive therapy					
Immunoglobulin administration, n (%)					< 0.001
No	8369 (95.88%)	8338 (97.49%)	8730 (98.03%)	8597 (98.49%)	
Yes	360 (4.12%)	215 (2.51%)	175 (1.97%)	132 (1.51%)	
Mechanical Ventilation, n (%)					< 0.001
No	5248 (60.12%)	4573 (53.47%)	4934 (55.41%)	4777 (54.73%)	
Yes	3481 (39.88%)	3980 (46.53%)	3971 (44.59%)	3952 (45.27%)	
Blood transfusions, n (%)					0.313
No	7896 (90.46%)	7699 (90.02%)	8090 (90.85%)	7902 (90.53%)	
Yes	833 (9.54%)	854 (9.98%)	815 (9.15%)	827 (9.47%)	
Outcome variable					
28-day mortality, n (%)					< 0.001
survival	6880 (78.82%)	7182 (83.97%)	7683 (86.28%)	7503 (85.95%)	
Non-survival	1849 (21.18%)	1371 (16.03%)	1222 (13.72%)	1226 (14.05%)	

English abbreviations: SOFA, Sequential Organ Failure Assessment; APACHE, Acute Physiology And Chronic Health Evaluation

### Results of univariate and multifactorial analyses of HGB and 28-day mortality

We investigated the relationship between HGB and 28-day mortality in patients with sepsis using various covariate correction procedures, and the results are shown in Table 2. In the unadjusted model, we found that for every 1-unit increase in HGB, the risk of 28-day mortality was reduced by 9% (odds ratio: 0.91, 95% CI: 0.90 to 0.93). After controlling for demographic characteristics, the risk of death within 28 days decreased by 9% for each unit increase in HGB (odds ratio: 0.91, 95% CI: 0.90 to 0.92). However, when adjusting for all covariates listed in Table 1, we observed that each unit increase in HGB was associated with only a 1% decrease in the likelihood of death after 28 days, and this association was not statistically significant (odds ratio: 0.99, 95% CI: 0.97 to 1.01).

**Table 2:** The findings of univariate and multivariable analyses

Hemoglobin vs. 28-day mortality	Non-adjusted model OR, 95%CI, P value	Adjust I model OR, 95%CI, P value	Adjust II model OR, 95%CI, P value
Hemoglobin (continuous variable)	0.91 (0.90, 0.93) < 0.0001	0.91 (0.90, 0.92) < 0.0001	0.99 (0.97, 1.01) 0.5056
Hemoglobin (Categorical variables (quratile))			
Q1	Reference	Reference	Reference
Q2	0.71 (0.66, 0.77) < 0.0001	0.69 (0.63, 0.74) < 0.0001	0.79 (0.71, 0.89) < 0.0001
Q3	0.59 (0.55, 0.64) < 0.0001	0.57 (0.52, 0.61) < 0.0001	0.78 (0.69, 0.87) < 0.0001
Q4	0.61 (0.56, 0.66) < 0.0001	0.60 (0.55, 0.65) < 0.0001	0.94 (0.83, 1.06) 0.3022
P for trend	<0.0001	<0.0001	0.0313

Non-adjusted model adjust for: None

Adjust I model adjust for: Gender; age at admission; Ethnicity/race

Adjust II model adjust for: Variables that are presented in Table 1

In order to perform sensitivity analysis, we transformed HGB into categorical variables based on quartiles and calculated p-values for trend tests. However, we found inconsistent results when comparing HGB as a continuous or categorical variable (Table 2). The non-linear association between HGB and 28-day mortality is suggested by the unequal variance in odds ratio values across the different hemoglobin groups. Therefore, our findings suggest a possible non-linear relationship between HGB and 28-day mortality in sepsis patients.

### Results of the non-linear association between HGB and 28-day mortality

**Fig. 2:**
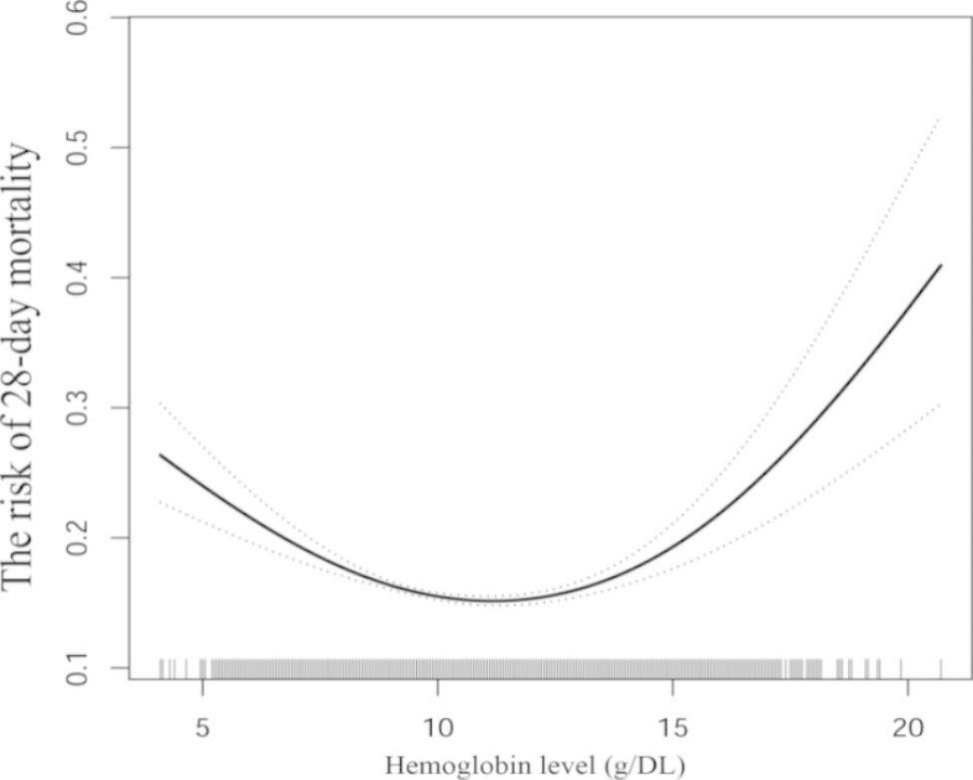
The association between HGB and 28-day mortality

To investigate the nonlinear relationship between HGB and 28-day mortality, we used smoothed curve fitting and generalized summation models. After controlling for the covariates presented in Table 1, our results (Fig. 2) showed a U-shaped relationship between HGB and 28-day mortality. We determined the inflection points using a two-stage linear model and recursive techniques, which were found to be at 104 g/L and 128 g/L. Our analyses indicated (Table 3) that when HGB levels were between 41 and 104 g/L, there was a 10% decrease in the risk of 28-day mortality (OR: 0.90; 95% CI: 0.87 to 0.94, p-value = 0.0001). However, in the range of 104–128 g/L, we did not observe a significant association between hemoglobin and 28-day mortality (OR: 1.17; 95% CI: 1.00 to 1.35, P value = 0.0586). When HGB was in the range of 128–207 g/L, there was a 7% increase in the risk of 28-day mortality for every 1 unit increase in HGB (OR: 1.07; 95% CI: 1.01 to 1.15, P value = 0.0424). These findings suggest that the relationship between HGB and 28-day mortality risk may not follow a linear trend, but instead exhibit a U-shaped pattern, with different ranges of HGB associated with varying levels of mortality risk.

**Table 3:** The addressing of nonlinear association between hemoglobin and 28-day mortality

	OR, 95%CI, P value
Fitting model using binary logistic regression model	0.99 (0.97, 1.01) 0.2176
Fitting model using two-piecewise linear model	
Inflection points	10.4, 12.8
<10.4	0.90 (0.87, 0.94) < 0.0001
≥10.4 to < 12.8	1.17 (1.00, 1.35) 0.0586
≥ 12.8	1.07 (1.01, 1.15) 0.0424
P for log likely ratio test	< 0.001

We adjusted for all covariates presented in Table 1

### Stratified analyses

We conducted a stratified analysis to investigate the trend of association between hemoglobin and 28-day risk of death in different subgroups of the population, using variables such as blood transfusion, GI bleeding, COPD, OSA, heart failure, and ischemic heart disease as stratification variables (Table 4). Our results showed that we did not observe any significant association between hemoglobin and 28-day risk of death, regardless of whether patients received transfusions or had OSA, heart failure, or ischemic heart disease (P for interaction was greater than 0.05). However, we found that in patients with combined GI bleeding (OR: 0.83, 95% CI: 0.72 to 0.94) and COPD (OR: 0.90, 95% CI: 0.84 to 0.96), a higher hemoglobin level was associated with a lower risk of death compared to patients without combined GI bleeding and COPD. There was a significant difference observed in P for interaction. These findings highlight the importance of considering the presence of comorbidities such as GI bleeding and COPD when evaluating the relationship between hemoglobin and mortality risk in sepsis patients.

**Table 4:** Use of blood transfusion or not as a stratification variable

	OR, 95%CI, P value	P for interaction
Blood transfusion		0.3154
No	1.00 (0.98, 1.02) 0.94381	
Yes	0.97 (0.91, 1.03) 0.3010	
GI bleeding		0.003
No	1.00 (0.98, 1.02) 0.8616	
Yes	0.83 (0.72, 0.94) 0.0037	
COPD		0.0012
No	1.00 (0.98, 1.03) 0.6747	
Yes	0.90 (0.84, 0.96) 0.0013	
OSA		0.3858
No	1.00 (0.97, 1.02) 0.7875	
Yes	0.97 (0.91, 1.03) 0.3109	
Heart failure		0.1136
No	1.00 (0.98, 1.03) 0.7669	
Yes	0.97 (0.94, 1.00) 0.0706	
Ischemic heart disease		0.4768
No	0.99 (0.96, 1.01) 0.2557	
Yes	1.00 (0.96, 1.05) 0.8378	

The adjustment strategy used in this analysis was similar to the Adjust II model, with the exception that the stratification variables were not adjusted for.

## Discussion

In this multicenter study, we examined the relationship between hemoglobin and 28-day mortality in patients with sepsis. Our analysis included a large sample of critically ill cohorts consisting of 34,916 individuals from the MIMIC-IV database. We found a U-shaped association between hemoglobin and the probability of 28-day mortality in patients with sepsis.

Our results indicated that there was a negative association between hemoglobin and the 28-day risk of death in patients with sepsis in the range of 41–104 g/L, and a positive association in the range of 128–207 g/L. Moreover, we observed that in the interval of 104–128 g/L, the risk of death of patients was the lowest. It is worth noting that despite significant efforts to discover new treatments and understand the causes of sepsis, mortality from sepsis has increased over the past 14 years. Our study highlights the importance of considering the hematologic system when evaluating patients with severe sepsis, as organ dysfunction is known to be associated with increased mortality in severe sepsis [23]. While previous studies have focused on the relationship between anemia indicators such as erythrocyte distribution width [24], hematocrit [15], and platelets [5, 13, 16, 17] and the prognosis of sepsis, we explored the potential nonlinear association between hemoglobin and 28-day mortality in septic patients.

Our study results are consistent with those of previous studies, such as the cohort analysis of 235 sepsis patients conducted by De Shengqi and Mei-Lin Peng [22], which showed that patients with early hemoglobin levels less than or equal to 80 g/L had significantly lower survival rates than those with levels greater than 80 g/L. Tan SMY et al. [10] confirmed similar findings in a cohort analysis of 8,132 sepsis patients, reporting that individuals with moderate anemia (hemoglobin 70 to 100 g/L) had worse prognosis than those without moderate anemia (> 100 g/L). Cai N et al. [25] found that a significant decrease in hemoglobin concentration was an independent risk factor for necrotizing small bowel colitis in preterm infants with sepsis. However, all of these studies reported only linear correlations, rather than non-linear correlations. In addition to establishing a negative association between hemoglobin and death, our study discovered for the first time that high hemoglobin levels are also associated with a significant risk of death. We hypothesize several reasons why this trend (higher hemoglobin associated with higher risk of death) was not found in previous comparable studies: (1) the population in our study consisted of adult critically ill patients in the United States, whereas previous studies with similar results studied populations in China or other regions; (2) our study had a relatively large sample size, making statistical analysis easier; (3) we used various nonlinear relationship algorithms, which have not been used in previous studies.

It is worth mentioning that Luo et al. [15] studied the relationship between hematocrit and 30-day mortality in sepsis patients using the same MIMIC-IV dataset. However, their results were different from ours: they found that mortality decreased with increasing hematocrit, rather than showing the U-shaped curve that we observed. The reason for this difference may be related to several factors: first, we had different extraction strategies for patient data. The sample size in our study was more than 10 times larger than that of Luo et al., and we extracted data using ICD-9 and ICD-10 codes (99,591–99,592, R652, R6520, and R6521), whereas they did not provide specific ICD codes used for patient selection. Additionally, our statistical models were significantly different from theirs: we used the GAM model for nonlinear associations and the two-piecewise linear model, whereas they did not use these models. Furthermore, it is important to note that Luo et al. used Cox regression, whereas we used logistic regression. We chose logistic regression because the follow-up time was short and consistent for all participants, minimizing the effect of time on the results.

The present study has several strengths. Firstly, it has a larger sample size than previous studies. Secondly, the study adjusts for more variables and has more stable results. Thirdly, the study uses algorithms that elucidate nonlinearity to better reflect the true relationship between hemoglobin and sepsis and 28-day death. Fourthly, to further explore the association between hemoglobin and the risk of 28-day death in different populations, we used diseases closely related to hemoglobin as stratification variables and found a trend where an increase in hemoglobin was associated with a lower risk of death in patients with GI bleeding and COPD. However, it should be noted that this is only an exploratory analysis and not corrected for type 1 errors caused by multiple comparisons, so the results need to be interpreted with caution.

However, this study has certain limitations: (1) Because this is an observational study, it is inevitably subject to confounding. However, we rigorously adjusted for confounding and used sensitivity analysis to assess the consistency of the results. (2) Due to the nature of observational studies, we can only observe the relationship between the two but not determine causality;(3) Because the database used in this study is a large single-center database with limited diversity, our results are subject to uniformity and may have unavoidable bias; (4) Because we can only adjust for measurable confounders but not for difficult-to-measure confounders, future clinical studies with higher levels of evidence in larger populations are required to validate our findings; (5) Since the immune status is closely related to the prognosis of sepsis, the use of immunosuppressive or chemotherapeutic agents could be a significant confounding factor. However, the MIMIC4 database did not include any information on such variables.

## Conclusions

In patients with sepsis, baseline hemoglobin was related to a U-shaped risk of 28-day death. When HGB levels were between 4.1 and 10.4 g/dL,there was a 10% decrease in the risk of 28-day mortality.However, in the range of 10.4–12.8 g/dL, we did not observe a significant association between hemoglobin and 28-day mortality.When HGB was in the range of 12.8–20.7 g/dL, there was a 7% increase in the risk of 28-day mortality for every 1 unit increase in HGB.

## Data Availability

The data that support the findings of this study are available from the corresponding author upon reasonable request.

## Abbreviations

SOFA: Sequential Organ Failure Assessment
APACHE: Acute Physiology And Chronic Health Evaluatio
